# Comparing Class-Aware and Pairwise Loss Functions for Deep Metric Learning in Wildlife Re-Identification †

**DOI:** 10.3390/s21186109

**Published:** 2021-09-12

**Authors:** Nkosikhona Dlamini, Terence L. van Zyl

**Affiliations:** 1 Faculty of Science, Braamfontein Campus, School of Computer Science and Applied Mathematics, University of Witwatersrand, Johannesburg 2000, South Africa; 2 Auckland Park Campus, Institute for Intelligent Systems, University of Johannesburg, Johannesburg 2006, South Africa

**Keywords:** Proxy-NCA, similarity learning, triplet-loss, semi-hard negative mining

## Abstract

Similarity learning using deep convolutional neural networks has been applied extensively in solving computer vision problems. This attraction is supported by its success in one-shot and zero-shot classification applications. The advances in similarity learning are essential for smaller datasets or datasets in which few class labels exist per class such as wildlife re-identification. Improving the performance of similarity learning models comes with developing new sampling techniques and designing loss functions better suited to training similarity in neural networks. However, the impact of these advances is tested on larger datasets, with limited attention given to smaller imbalanced datasets such as those found in unique wildlife re-identification. To this end, we test the advances in loss functions for similarity learning on several animal re-identification tasks. We add two new public datasets, Nyala and Lions, to the challenge of animal re-identification. Our results are state of the art on all public datasets tested except Pandas. The achieved Top-1 Recall is 94.8% on the Zebra dataset, 72.3% on the Nyala dataset, 79.7% on the Chimps dataset and, on the Tiger dataset, it is 88.9%. For the Lion dataset, we set a new benchmark at 94.8%. We find that the best performing loss function across all datasets is generally the triplet loss; however, there is only a marginal improvement compared to the performance achieved by Proxy-NCA models. We demonstrate that no single neural network architecture combined with a loss function is best suited for all datasets, although VGG-11 may be the most robust first choice. Our results highlight the need for broader experimentation and exploration of loss functions and neural network architecture for the more challenging task, over classical benchmarks, of wildlife re-identification.

## 1. Introduction

Wildlife monitoring and re-identification have evolved from capture-recapture, radio frequency identification to image-based automated re-identification using animal biometric features. Capture-and-recapture was dominant in the early to late 1990s when population estimation experiments extended over a year; first, animals were captured and marked then recaptured periodically to obtain a count [1]. Capture-recapture poses challenges where animal/human confrontation is unnecessary when the studies involve animals such as Lions. Ariff and Ismail [2] state that the RFIDs technology emerged as an alternative since using RFIDs removes the need to recapture. Individuals can, as a result, be identified from a distance. However, Schacter and Jones [3] discuss that implanting RFID tags alters the physiology and behaviour of the host animal. These behavioural changes can lead to ill-health and other effects that negatively impact the conservation of wild animals. Further, RFIDs have high costs associated with the deployment and maintenance of these systems [4]. Recent studies aim to replace RFID technologies with image-based biometric systems [5].

Animal biometric re-identification using images originated in domesticated animal applications and has gradually transitioned towards wild animal species [6]. Research in image-based biometric re-identification of animals was prompted by recent advances in human biometric projects using deep neural networks [7]. However, often images of wild animals are occluded by tree branches, contain background or are intentionally camouflaged [8]. The work of Verma and Gupta [9] shows that the deep neural networks can detect the animal existence in an image in what is termed animal background verification.

The era of image-based biometric re-identification is dominated by a search for better neural network architectures, better parameters, and better approaches ranging from deep neural networks for classification to deep neural networks for similarly learning, with the latter being the main focus of recent research. Deep neural networks for similarity learning involve selecting pair/triplet sampling techniques, ranking loss functions, and model evaluation metrics. However, these approaches are often evaluated on larger datasets with thousands of data points and tens of instances per individuals/class, all relatively well-balanced [10,11]. As a result, there remains a gap in knowledge concerning the efficacy of the increased complexity of recently proposed methods on smaller unbalanced datasets with fewer instances per class. The survey by Kaya and Bilge [12] depicts a summary of these advances in similarity learning and discusses varying model performances on different datasets.

The current research investigates how different design choices in deep similarity learning affect model performance on fine-grained biometric re-identification tasks in wild animals. Specifically, the current research focuses on comparing recent advances in loss functions and neural network architecture. The models are evaluated using Recall@1 and mean average precision at *R* (MAP@R) [13]. Our main contribution is to demonstrate that there is no single neural network architecture combined with a loss function that is best suited for animal re-identification. In particular, we:

- highlight the need for increased research into loss functions and neural network architecture specifically for wildlife re-identification;
- improve on the state-of-the-art results in numerous animal re-identification tasks;
- contribute two new benchmark datasets with results;
- provide minor support for a choice of triplet loss with a VGG-11 backbone as an initial architecture and loss.

## 2. Related Work

### 2.1. Animal Biometrics and Computer Vision

Wildlife biometric identifiers are distinctive and measurable characteristics used to label and describe individual animals within the population. Animal biometrics is the field that studies visual and behavioural features that discriminate species and individuals within species in the animal kingdom [14]. Identifying species is a coarse-grained task using mutual features across individuals, whereas individual re-identification is a fine-grained task reliant on distinct features among individuals. Fine-grained re-identification requires expert knowledge to distinguish between individuals in a species who might share many mutual features [15]. According to Kühl and Burghardt [16], the features used varies among species since a specific set of criteria needs to be met before features become a biometric feature candidate, namely [17]:

**Universality:** all the individuals in the population must have such a feature;
**Uniqueness:** two or more individuals should have a different form of the same feature.

The use of biometric re-identification through images to identify animals became popular because there are few disturbances in animals’ natural habitat as the animal image data are collected via camera traps. Computer vision experts researched different areas in the systems developed for re-identification through images [18]. The broader areas of research included improving model deployment flexibility and overall experiment design, from the sampling of training data points up to more robust ways of evaluating model performance.

### 2.2. Classification vs. Similarity Learning

According to Krizhevsky et al. [19], two main approaches employed in computer vision using deep convolutional neural networks (DCNN) are classification and similarity learning. Chen et al. [20] and Li et al. [21] demonstrate that (DCNN) produced state-of-the-art performance in solving ImageNet data tasks. Systems that emerged from classification-driven tasks were DeepID [22] which achieved an accuracy of 99.15% in human re-identification using face images. In classification tasks, the top layers of the DCNNs are responsible for assigning *N*-way class probabilities via an activation function, namely: soft-max or sigmoid. Models trained for *N*-way classification cannot be used if the number of classes in the dataset increases, retraining the model becomes necessary, this reduces the real-world deployment of these models as training deep neural networks may take several days [23]. As a result of this, there is an interest in finding ways of avoiding costly retraining, and research on similarity learning models has gained traction [24].

### 2.3. Similarity Learning

Similarity learning does not include an output layer that assigns class probability [25]. Instead, a similarity models’ output layer produces an embedding of the input image into a representative feature space. A similarity learning model has two identical neural networks called Siamese neural networks. These networks are trained with back-propagation to optimise a ranking loss function. Each neural network produces an embedding through back-propagation, and the network’s weights are updated to minimise the loss [26].

A similarity learning model is trained to learn a representative feature vector of an object from an image signal. The feature vector is generated so that dis/similar real-world objects are dis/similar in the embedding space. The application of similarity learning dates back to early 1990s where Bromley et al. [27] used similarity learning in automating signature verification [28]. Schroff et al. [29] discussed the successes of similarity learning in the re-identification of human beings using facial images. There are a significant number of loss functions adopted in experiments for training similarity learning models, namely contrastive loss, triplet loss, and Proxy-NCA [30,31,32].

### 2.4. Sampling Techniques for Pairwise Training

Schroff et al. [29] contended that for effective training of similarity learning models on triplet loss, it is essential to select meaningful pairs/triplets. Several research efforts produced the following pair mining schemes:

#### 2.4.1. Hard Negative Mining

Hard negative mining is selecting image pairs; $x_{a}$ and $x_{n}$ that have most similar embedding vectors, yet they belong to different classes $C\left(x_{a}\right)\neq C\left(x_{n}\right)$:

(1) $$x_{s}=\underset{d}{\mathrm{argmin}}\;d\left(f\left(x_{a}\right),f\left(x\right)\right)x:C\left(x_{a}\right)\neq C\left(x\right),$$

where $x_{s}$ is the candidate sample, *f* is a function that produces the embedding vectors for both $x_{a}$ and $x_{n}$, and *d* is a distance measure [33]. The measure of how close two embedding vectors are is based on the hyper-parameter: margin or α. Where a triplet selection is needed, it is expected that the distance between an anchor image and a positive image be less than the distance between anchor image and negative image:

(2) $$d(f\left(\left(x_{a},f\left(x_{p}\right)\right)\right)+\alpha \;<d\left(f\left(x_{a},f\left(x_{n}\right)\right)\right).$$

#### 2.4.2. Semi-Hard Negative Mining

Schroff et al. [29] argue that the Hard-negative pair mining technique can lead to bad generalisation and model collapse. An alternative to Hard-negative pair mining was proposed; known as Semi-hard negative, where the distance between the anchor and negative example images is allowed to lie within the margin α but still far from the distance between positive and anchor examples Equation (3):

(3) $$d(f\left(\left(x_{a},f\left(x_{p}\right)\right)\right)<d(f\left(x_{a},f\left(x_{n}\right)\right).$$

The semi-hard negative triplet mining and triplet loss minimisation produced superior performance in human re-identification from face images [29]. Several additional mining techniques include easy positive mining, easy negative mining, and hard positive mining. Details on these techniques are discussed by Xuan et al. 2020 [33].

Previous works have shown that even with the best performing architecture, these mining techniques and loss functions are not consistent across all datasets. The DenseNet-201 neural network produced 75.5% for the chimpanzee dataset and 92.2% accuracy for the octopus dataset [34]. Another observation is that researchers decide on different metrics to measure their models’ performance; some report accuracy, others Recall@*k*, and some mean average precision MAP, making it challenging to compare [12,35].

### 2.5. Challenges in Deep Metric Learning

Roth et al. [35] highlighted the challenges of comparing works in metric learning. These challenges are a result of the varying objectives the researchers are addressing in their work. Some researchers are seeking to improve the architecture setup [36], others are aiming to obtain better objective functions (loss functions), while others introduce new algorithms to solve problems. Kim et al. [37] proposed an efficient facial expression recognition algorithm. However, it is difficult to ascertain that the improvements come from the proposed algorithm or design choices, namely: feature extraction methods, choice of optimiser function or the selected network architecture. The concerns expressed by Musgrave et al. [13] are to the effect that experiments should be compared fairly by isolating the contributing factor to the observed performance improvements. One way to isolate the contributing factor is by applying the same algorithm to different neural network architectures across various animals to confirm that improvements come from the proposed factor.

Musgrave et al. [13] clarified that before metric learning projects claim superior performance over previous works, there should be a fair comparison. This comparison includes keeping all parameters the same to identify what contributes to the improvement. Comparing experiments where different neural network architectures and different model parameters are used is seen as problematic. Roth et al. [35] and Schroff et al. [29] agree that changing the embedding dimension size has an effect on model performance. Similar observations were made by Wang et al. [38] where the embedding sizes were tuned to find an optimum size that results in good performance while keeping all parameters of the model the same. This discussion highlights that a simple change of the output embedding size can create an unfair comparison if the objective was to compare, say, for instance, loss functions.

Another area that can create unfair comparisons is selecting loss optimiser functions and their hyper-parameters, such as the learning rate. It may not be incorrect to find a neural network architecture, an optimiser, and hyper-parameters that yield good performance in solving a task. However, a requirement should be transparency in the choices and omissions made by researchers. If future works make similar comparisons, the objectives need to be stated, and all other design choices must be kept consistent.

### 2.6. Animal Biometrics Using Image Features

Deep metric learning has received significant attention for solving image-based problems. The gradual use of neural networks on wild animals datasets is reflected in the survey conducted by Schneider et al. [39]. In the re-identification of animals, van Zyl et al. [8] used deep metric learning to identify individuals in Zebra and Nyala datasets. ResNet-50 pre-trained on ImageNet dataset was used as the DCNN backbone architecture, the contrastive loss approach was employed to output embedding vectors of 40 dimensions. The training pairs were randomly sampled. The datasets used in the experiments were comparatively smaller: 820 Zebra data points of 84 classes (individuals), and the Nyala was 1945 data points of 474 classes. Schneider et al. [34] presented a comparative study between AlexNet, VGG-19, DenseNet-201, ResNet-152 and Inception-V3 in re-identification of Chimpanzees, Whale, Fruitfly and Tiger. The mean average precision achieved by DenseNet-201 DCNN trained on triplet loss in the Chimpanzees dataset was 93.2%.

Qiao et al. [40] used the Inception-V3 convolutional neural network to extract discriminating features from images of farm cattle. A Long short term memory (LSTM) was used to extract temporal features from video sequences that capture variations in gait among other temporal features of an individual in the cattle dataset. The Inception-V3 was pre-trained on ImageNet. There were 8780 images of 41 individuals. The classification layer was reduced to 41-dimensions, representing the number of classes in the dataset. The loss function used during training was categorical cross-entropy loss.

Other researchers looked at the re-identification of individuals from Panda face images. Hou et al. [41] collected and experimented on 25 individual Pandas each with about 4300 images. The total number of data points was 65,000. VGGNet was trained with the top fully connected layer of 25-dimension, a soft-max activation function was used to assign class probabilities. The reported metric is the accuracy of 95% for detecting individuals from the test dataset and 78.7% on detecting unknown individuals in the dataset.

Korschens and Denzler [15] investigated the use of ResNet-50 neural network architecture, pre-trained on ImageNet, in the re-identification of individuals in an elephant dataset. Earlier layers of ResNet-50 were fine-tuned instead of fine-tuning the top layer. The features generated by ResNet-50 were used to fit an SVM classifier model. The elephant dataset had just over 2000 data points split into 75% training and 25% test set. The reported top-10 accuracy was 79%. Korschens and Denzler [15] did not investigate shallower variants of the ResNet architecture or alternative convolutional neural network architectures.

Nepovinnykh et al. [42] adapted a Deeplab [20] model that was trained on triplet loss to re-identify individuals in a ringed seals population. Deeplab was pre-trained on Pascal VOC dataset [43], no pair sampling technique was discussed in the Deeplab system. The top-5 accuracy reported was 87%. The accuracy reported was on a hold-out test data of 2000 points, with 46 individual seals.

The current work expands on previous wildlife re-identification by using a similarity learning approach coupled with searching for optimal loss functions, sampling techniques, and convolutional neural networks architecture. The specific species considered are individuals in a lion population and individuals in a Nyala population. The only prior study to the authors’ knowledge working on a Lion dataset focused on annotating lion images to depict the lion’s activity [44].

Further, we note the threat to the world population of Lions demands that conservation efforts be enhanced [45]. Norouzzadeh et al. [46] stated that conservationists will leverage automated counting and tracking animals to report on biodiversity reliably, with less human effort and fewer disruptions on the habitat of wild animals.

### 2.7. Loss Functions

#### 2.7.1. Pairwise Loss

Contrastive loss is the original pairwise loss function that takes a pair of image samples, and a class indicator function. The training follows a feed-forward with back propagation, learning embedding vectors that maximise the embedding distance if the images are from a different class, and minimises the distance if the images belong to the same class [30].

**Figure 1:**
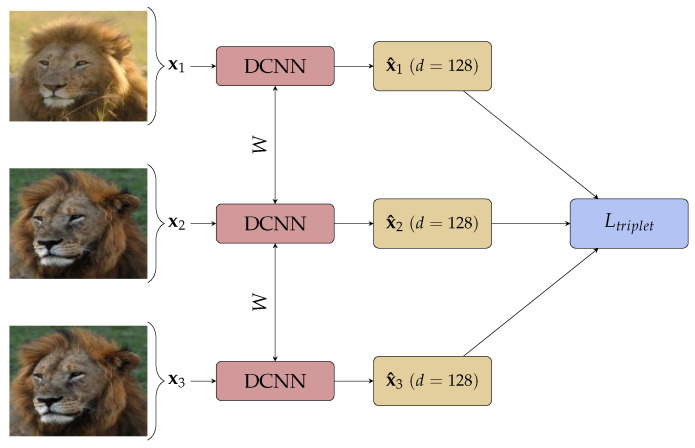
Triplet Network with *d* the dimension of the embedding.

Triplet loss, an improvement on contrastive loss shown in Figure 1, takes three images: an anchor image $x_{i}^{a}$, a positive image $x_{i}^{p}$ and a negative image $x_{i}^{n}$. The network learns to minimise the Euclidean distance between the embedding of anchor and positive image, and increases the distance between the anchor and negative image simultaneously by minimising:

(4) $$\sum_{i}^{N}\left\lceil \|f\left(x_{i}^{a}\right)-f\left(x_{i}^{p}\right){\|}_{2}^{2}-{\left\|f\left(x_{i}^{a}\right)-f\left(x_{i}^{n}\right)\right\|}_{2}^{2}+\alpha ,0\right\rceil ,$$

where α is a hyper-parameter forcing a minimum distance between images of different class. A graphical view of the triplet loss training is given in Figure 1.

The triplet loss and contrastive loss functions are regarded as pairwise losses since they are computed considering the similarities of pairs. The triplet loss approach, coupled with semi-hard pair sampling, resulted in state-of-the-art performance in human face re-identification in experiments [29]. As reflected in Section 2.6, triplet loss and contrastive loss have been applied in animal re-identification tasks by Nepovinnykh et al. [42] and van Zyl et al. [8]. The pairwise loss functions demand that meaningful pairs are found during the training phase. Due to the inefficiencies of pair-mining, researchers explored ways to avoid mining training pairs by developing class-aware loss functions [47].

#### 2.7.2. Class Distribution Based Loss

Wang et al. [48] and Chen et al. [49] also acknowledged the limitations of pairwise loss functions such as contrastive loss; however, instead of formulating a completely new loss functions, they proposed a distribution aware variant of the contrastive loss function. Rippel et al. [50] proposed a deviation from pairwise losses to what Wang et al. [38] termed class aware losses. The proposed loss function, Magnet loss, considers class clusters that are updated during training. These clusters capture intra-class variations but also penalise inter-class overlaps. This approach ensures that clusters of the same class attract and clusters of different classes repulse. This approach is different from the pairwise loss functions that do not consider clusters but individual pairs. Rippel et al. [50] note that the Magnet loss function demands that partially pre-trained models be used; through fine-tuning deeper layers as opposed to fine-tuning top layers.

A similar approach of deviating from pairwise losses to class-aware losses is discussed by Movshovitz-Attias et al. [51]. An adaptation of NCA [52] is presented called Proxy-NCA, with a deliberately constructed small set of data points *P* referred to as proxies to a point *x*. The assumption is that a point in *P* is sufficiently close to *x* in terms of a distance *d* and as such can be a substitute for *x*. This point called the proxy of *x* is given by:

(5) $$p\left(x\right)=\underset{p}{\mathrm{argmin}}\;d\left(x,p\right),$$

where p∈P. ϵ is the proxy approximation error and is given by the maximum error using all data points in *P*:

(6) $$\epsilon =\max\;d\left(x,p\left(x\right)\right).$$

In the case where class labels are available, the proxies are selected using class labels. The ranking loss is minimised amongst a data point anchor *x* with two proxies p(y), p(z) where p(y) is a proxy of *x* with data points of the same label, and p(Z) is a proxy of the data point with a different label from the anchor. In each training iteration, sample *P* containing (x,y,Z) is selected from the training dataset, and

(7) $$l=-\log\left(\frac{\exp\left(-d\left(x,p\left(y\right)\right)\right)}{\sum_{p\left(z\right)\in p\left(Z\right)}\exp\left(-d\left(x,p\left(z\right)\right)\right)}\right)$$

is minimised. This approach removes the need to mine training pairs. Proxy-NCA was used with inception network architecture in image retrieval tasks for datasets: Cars196 [53] and Stanford Products dataset, where better performance is observed with a margin improvement of 21.7% when compared with the same model trained on triplet loss and semi-hard mining technique [51]. Teh et al. [47] adapted the Proxy-NCA to Proxy-NCA++ which incorporates maximising a proxy probability instead of minimising a proxy distance. Increasing the proxy probability has the same effect of attracting a data point to its positive data points proxy-set and repels data points of different proxies. Teh et al. [47] further proposed a scaling factor to be applied in the proxy loss function in order to scale the distribution of probability across classes. We do not consider Proxy-NCA++ further in this research.

### 2.8. Motivation

The emerging loss functions that are aimed at replacing the pairwise loss are tested on larger datasets such as Stanford Cars [54] and CUB-200 [55]. The effectiveness of the proposed class-aware loss functions remains unknown for smaller, highly unbalanced datasets. We compare models trained on class-aware loss function with models trained on traditional pairwise loss functions. To provide fair comparisons, we conducted experiments taking into account the challenges identified in Section 2. We widen the scope of our experiments by investigating the effect of changing the loss function across six datasets, applied to five different neural network architectures.

## 3. Materials and Methods

**Figure 2:**
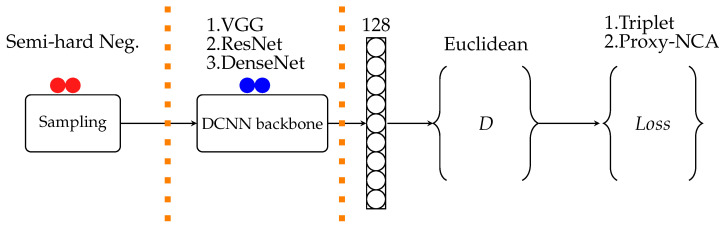
Experiment setup starts with training pairs selection; then, DCNN is the network architecture chosen for a particular experiment, the output of DCNN is an embedding vector of dimension *D* and distance metric measured with ranking loss function being minimized.

The structure of experiments followed in deep neural networks projects is depicted in Figure 2. The boundaries indicate areas where design choices are made that affect the resulting models’ performance. The sampling section is only relevant when pairwise loss functions are used, such as triplet loss. We ran each experiment 10 times for the train and test set. Each training experiment consists of 30 epochs, and one epoch uses 5-fold cross-validation to update the model weights. Our experiments are compared using two loss functions: Triplet-loss and Proxy-NCA, on six animal datasets discussed further below.

### 3.1. Data

We carry out experiments using Lion face data collected from the Mara Masia project in Kenya (http://livingwithlions.org/mara/browse/all/all/) (accessed on 27 October 2019). We also trained our models on the Nyala dataset collected from South African nature reserves. Both of these datasets, Nyala and Lions have relatively few (7) examples per an individual. In addition, we conducted experiments with publicly available datasets, namely Tai Zoo Chimpanzee [56], Zebra [57], Panda [58] and Tiger [59] datasets to compare our approach with previous works. Table 1 shows the total data points each dataset has, total individuals in the dataset, and average samples per individual. The neural network architectures we trained were VGG, ResNet, and DenseNet. We followed a zero-shot train-test split, such that all classes in the test set did not form part of the training set.

### 3.2. Neural Network Architectures

The VGG network architecture came as an improvement to a neural network architecture developed by Krizhevsky et al. [19]. Simonyan and Zisserman [60] constructed VGG as a stack of convolutional layers that use smaller filters of [3×3] size as opposed to the previous neural networks that used filters of varying sizes. In the VGG network, all hidden layers have a ReLU activation function to induce non-linearity. Three fully connected layers, two of these layers have a dimension of 4096, and the last layer with dimension 1000 to capture the classes found in the ImageNet dataset. The Soft-max activation function is applied to the last layer to capture class probabilities. The VGG comes in two commonly used variants, and one variant has 11 layers (VGG-11), eight convolutional layers, and three fully connected layers. VGG-19, on the other hand, is 19 layers deep (16 convolutional layers and three fully connected layers).

He et al. [61] proposed a different convolutional neural network setup (ResNet) to overcome vanishing gradients. He et al. [61] suggested that instead of having stacked layers, some deeper layers are skipped. The motivation for skipping is based on the assumption that if the deeper layers suffer from a vanishing gradient, then earlier layers should have learned an optimal mapping. The output of the earlier layers is used to learn a residual mapping, and this mapping is added on deeper layers preserving the information learned before a possible vanishing gradient occurs. The ResNet architecture comes in numerous variations, namely: ResNet-34, ResNet-50, ResNet-101 and ResNet-152, where the number indicates total layers in the residual neural network.

Huang et al. [62] suggested DenseNet, which is an alternative of both VGG and ResNet architectures. VGG and ResNet are based on the premise that a prior layer’s output is fed into the next layer as input. DenseNet, on the other hand, suggests a densely connected architecture where the next layer is fed with output from all other preceding layers. The positive effect of having densely connected neural layers is that the dense connections have a regularisation effect, a good thing for smaller datasets where over-fitting is prevalent. DenseNet also comes in several variants, such as DenseNet-121, DenseNet-201. It is worth noting that the number of layers is not fixed to 121 and 201. Some DenseNet can have *N* layers and are referred to as DenseNet-*N*.

### 3.3. Training

We split our data into 80% training and 20% test set. Table 1 contains the total data points in both the train and hold-out test set. The 80% train set was split into 5-folds in each training epoch. All the backbone architectures used were pre-trained on ImageNet, and these were used as feature extractors as opposed to fine-tuning all layers. We replaced the 1000-class output layer from ImageNet with a 128-dimensional embedding layer for one set of our experiments. On another set, we searched for an optimal output layer size.

We trained for 30 epochs minimising the Proxy-NCA loss. The Proxy-NCA loss parameters need an optimiser; we used the Adam optimiser with default parameters $\beta_{1}=0.9$, $\epsilon =10^{-3}$ and $\beta_{2}=0.999$. The learning rate was set to 0.0001. The same parameters were used in triplet loss experiments. However, the additional parameter in triplet loss, the margin was kept at 0.2. A semi-hard negative sampling technique was used on batch examples selected randomly from the training set. We ran ten train-test iterations and the results reported are the average performance metrics of the ten test runs with confidence intervals as done in [13].

### 3.4. Metrics Measured

A recently developed benchmark PyTorch library [63] was used to measure all the metrics reported in our experiments. During the model evaluation, we used hold-out test data containing classes that were not seen during the training phase. To compare with the previous works, we computed the Recall@1. We also measured the mean average precision at *R* (MAP@R) given in Equation (8) by:

(8) $$MAP@R=\frac{1}{R}\sum_{i=1}^{R}P\left(i\right),$$

where P(i) is precision at *i* if the *i*th neighbour is a true positive otherwise P(i) is 0. MAP@R is the mean average precision computed over *R* nearest neighbours of the query image retrieved by the model of Musgrave et al. [13]. We did not find papers that reported on MAP@R for the datasets we used in our experiments.

### 3.5. Embedding Dimension Size

For each dataset, we verified if using 128-dimension embedding for the output layer is justified. We tested three embedding dimension sizes at 64, 128 and 512. We kept all other network parameters constant, from sample size up to the loss function parameters and only altered the dimension of the layer before the output layer. The results for each dataset with varying embedding layer dimensions are shown in Table 2 and justify the use of 128 across all experiments.

## 4. Results

As reflected in Table 2, for each dataset, we run preliminary experiments where we searched for the best size of the output embedding vector that results in improved model performance. All the results we present were generated using optimal embedding size for each dataset as found from the preliminary investigation. We present our results for the neural network architectures and datasets used in the experiments. We run a pair of experiments for each neural network backbone: triplet loss vs. Proxy-NCA loss across all datasets. The aim is to observe the effect of each loss function when the dataset, network backbone, and parameters are kept constant.

Table 3 presents Recall@1 and Table 4 presents mean average precision at *R* (MAP@R). In our tables, we further show what body parts the images of the individual animals contained. The Lions, Panda, and Chimpanzees datasets have faces, while Nyala, Tiger, and Zebra datasets contained the body side (flanks). We report the average of ten train-test runs with statistical significance obtained by doing a two-sided *t*-test at 95% confidence level. The results in bold in Table 3, and Table 4 are the best performing method. In the same tables, the results in grey are those that are not statically significantly different from the best. Table 5 is extracted from Table 4 where we highlight the best-performing methods concerning both the mean and standard deviations.

## 5. Discussion

### 5.1. Class Aware vs. Pairwise Loss

The observations made from our results are that the pairwise loss function performs better than the class aware loss function. However, the difference in performance between the loss functions is marginal. This trend is observed across all the datasets and neural networks we investigated. To illustrate this point, DenseNet-201 trained on triplet loss achieved the best performance of 79.7% on the Chimpanzees dataset, while DenseNet-201 trained on Proxy-NCA on the Chimpanzees dataset obtained 78.2%. The second trend is that the best performance is not always achieved by the same neural network architecture and loss function across all datasets. For the Tiger and Panda datasets, VGG-11 trained on triplet loss achieved 88.9% and 91.2%, respectively. In the Lion dataset, VGG-19 Proxy-NCA obtained the best performance of 71.3%. Musgrave et al. [13] who studied three datasets Cars-196, CUB-200 and Stanford online products made the same observations. While we cannot directly compare our work with the results presented by Musgrave et al. [13], we also found that it is not always that Proxy-NCA will outperform the pairwise loss function.

**Figure 3:**
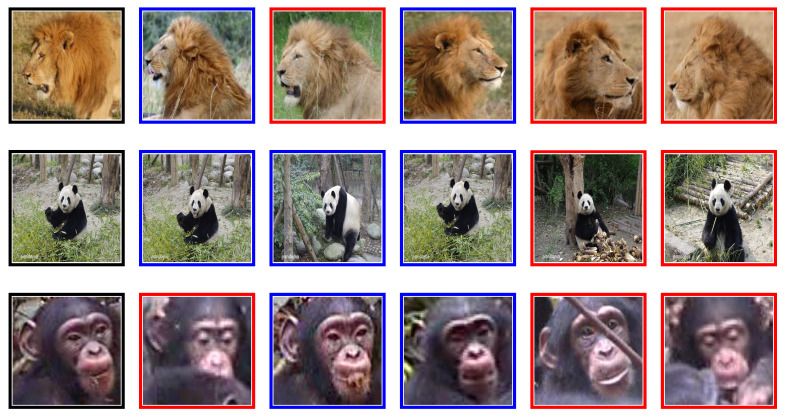
Faces: The first image is the query, with five nearest neighbours: blue border is true positive and red border is false positive image.

**Figure 4:**
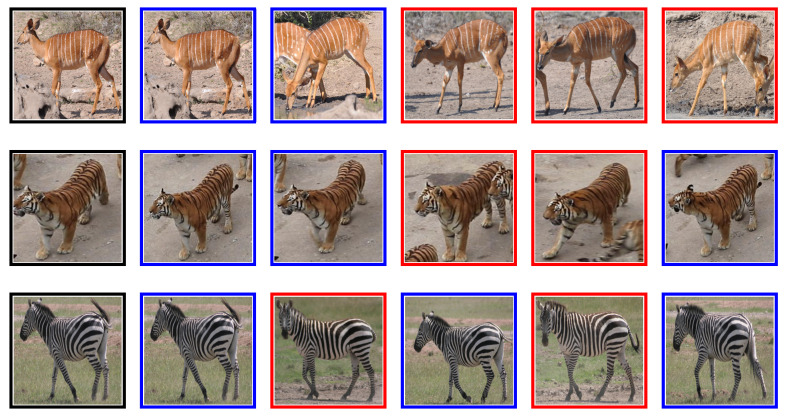
Flanks: The first image is the query, with five nearest neighbours: blue border is true positive and red border is false positive image.

A study carried out by Chen et al. [64] on the Panda dataset obtained Recall@1 of 92.1% on unseen classes. However, a major preprocessing step involved hand annotation locating the Panda face in the images, so that background and occlusions are removed from each image before using the image for training. While the proposed algorithm yielded good results, we demonstrate that VGG-11, trained on triplet loss function, compares well with the method proposed by Chen et al. [64]. VGG-11 triplet loss obtained 91.2 ± 1% on raw Panda images depicted in Figure 3, without a need to apply extensive preprocessing steps. We observe that removing background may create challenges when identifying Pandas in their natural habitat. On the Tiger dataset, Schneider et al. [34] found that DenseNet-201 triplet loss achieved a Recall@1 of 86.3%, we replicated these results, our DenseNet-201 obtained 85.0% ± 1; however, we found that VGG-11 trained on triplet loss performs better at 88.9%. A sample of retrieved images from animal flanks is shown in Figure 4.

### 5.2. Backbone Architectures

Investigating the best performing backbone architectures was not the primary objective of the research. Still, it is interesting to note from Table 4 that if one were to select a single backbone, then VGG-11 with Triplet loss would give one the most consistently robust results followed by DenseNet-201 also with triplet loss.

### 5.3. Flanks vs. Faces

We test out various architectures in combination with different loss functions and different embedding sizes to see if some generalised lessons exist for animal faces versus flanks. We find no obvious commonality, which leads us to hypothesise that perhaps pre-training on ImageNet was sufficient. To this end, we explored fine-tuning the models using datasets on flanks for flanks and faces for faces but found no benefit for transfer learning. Further, we find that using more advanced/complex or suited towards faces Neural Network architectures leads to no statistically significant improvements.

## 6. Conclusions

Recent works in metric learning have focused on loss function design, intending to substitute the pairwise loss functions. Comparing these loss functions across datasets in individual re-identification of wild animals; Lions, Chimpanzees, Zebra, and Nyala, we find that the performance gains are marginal. More often, the triplet loss models produced better results.

Our work shows the proposed improvements in the design of deep neural networks for similarity learning experiments. From how training samples are selected to how loss functions are optimised, there cannot be one solution that is best suited for all datasets. Each dataset presents a unique challenge to the model, and hence, there is a need to look for the best possible design for each dataset continuously. The models trained on the triplet loss function achieved better Recall@1 across datasets. It is worth pointing out that the Proxy-NCA loss removes the need to search for an optimal sampling technique. This removal cannot be overlooked as it simplifies experimental design. In the future, it may be necessary to investigate a combination of model parameters and a specific loss function to improve models’ information retrieval capabilities.

## Figures and Tables

**Table 1:** Sample Size (*N*), Individuals (*I*) and Mean Examples (#) per Individual in Full and Train/Test Sets.

Animal	*N*	*I*	E[#/I]	Split	*N*	*I*
**Lion**	750	98	7.7 ± 4.0	**Train** **Test**	594 156	79 19
**Nyala**	1934	274	7.1 ± 5.1	**Train** **Test**	1213 729	179 95
**Zebra**	2460	45	54.7 ± 7.3	**Train** **Test**	1989 471	36 9
**Chimp**	5078	78	65.1 ± 17.0	**Train** **Test**	3908 1170	62 16
**Panda**	6462	218	29.64 ± 8.0	**Train** **Test**	5546 916	174 44
**Tiger**	3651	182	20.1 ± 15.0	**Train** **Test**	1887 1764	107 75

**Table 2:** DenseNet-201 Proxy-NCA loss, performance search for optimal output embedding dimension D.

	MAP@R%		
Dataset	D-64	D-128	D-512
Chimp	8.4 ± 0	9.1 ± 1	9.1 ± 0
Nyala	38.0 ± 1	38.6 ± 1	38.5 ± 1
Zebra	29.8 ± 2	29.6 ± 1	30.6 ± 2
Lion	48.8 ± 2	50.6 ± 1	50.5 ± 2
Tiger	21.6 ± 3	23.2 ± 2	23.0 ± 3
Panda	27.5 ± 1	28.4 ± 2	28.1 ± 1

**Table 3:** Recall@1: triplet loss semi-hard mining vs. Proxy-NCA for training classes. Bold indicates the best performing method, and grey highlights results that are not statistically significantly different from the best.

		Top-1/Recall@1					
		Faces			Flanks		
Architecture	Loss	Lions	Chimps	Pandas	Nyala	Zebra	Tiger
VGG-11	Triplet	66.5 ± 2	79.0 ± 1	**91.2 ± 1**	68.7 ± 2	94.6 ± 0	**88.9 ± 1**
	P-NCA	68.2 ± 3	78.9 ± 1	89.3 ± 2	68.4 ± 2	93.8 ± 2	87.0 ± 1
VGG-19	Triplet	70.2 ± 2	70.6 ± 0	86.3 ± 2	**72.3 ± 0**	82.8 ± 1	86.3 ± 2
	P-NCA	**71.3 ± 3**	66.3 ± 0	90.9 ± 0	69.2 ± 3	82.7 ± 0	84.4 ± 1
ResNet-18	Triplet	67.8 ± 1	79.2 ± 2	90.0 ± 0	64.9 ± 2	**94.8 ± 1**	87.1 ± 1
	P-NCA	66.8 ± 3	77.9 ± 0	90.1 ± 1	64.1 ± 0	93.6 ± 2	84.8 ± 1
ResNet-152	Triplet	63.2 ± 2	71.2 ± 1	87.6 ± 3	61.0 ± 3	80.7 ± 0	76.5 ± 2
	P-NCA	61.0 ± 1	69.5 ± 1	83.4 ± 0	59.7 ± 0	79.1 ± 3	75.5 ± 2
DenseNet-201	Triplet	70.1 ± 1	**79.7 ± 2**	89.6 ± 1	67.1 ± 2	89.1 ± 0	85.0 ± 1
	P-NCA	69.5 ± 3	78.2 ± 2	90.7 ± 1	66.3 ± 1	87.5 ± 0	85.6 ± 1
Prior Research	-	-	77.5 ± 0	92.1 ± –	72.1 ± 0	72.6 ± 0	86.3 ± 0

**Table 4:** MAP@*R*: triplet loss semi-hard mining vs. Proxy-NCA on training classes. Bold indicates the best performing method, and grey highlights results that are not statistically significantly different from the best.

		MAP@R					
		Faces			Flanks		
Architecture	Loss	Lions	Chimps	Pandas	Nyala	Zebra	Tiger
VGG-11	Triplet	16.5 ± 2	12.9 ± 2	**32.0 ± 2**	**11.2 ± 0**	16.8 ± 1	22.8 ± 1
	P-NCA	17.7 ± 1	**13.8 ± 3**	31.8 ± 1	11.0 ± 1	16.5 ± 0	22.9 ± 2
VGG-19	Triplet	18.0 ± 2	11.7 ± 1	25.0 ± 0	10.8 ± 1	16.7 ± 2	21.8 ± 1
	P-NCA	17.7 ± 0	12.0 ± 2	28.7 ± 0	9.7 ± 3	16.4 ± 3	20.0 ± 1
ResNet-18	Triplet	18.5 ± 0	11.2 ± 2	26.3 ± 1	9.9 ± 2	**19.0 ± 0**	**24.6 ± 4**
	P-NCA	19.0 ± 1	11.5 ± 1	24.9 ± 0	9.5 ± 1	18.2 ± 1	21.7 ± 2
ResNet-152	Triplet	17.3 ± 2	10.1 ± 0	26.9 ± 1	8.2 ± 0	12.1 ± 3	12.5 ± 3
	P-NCA	17.1 ± 0	9.4 ± 3	20.3 ± 1	9.0 ± 2	11.9 ± 2	11.0 ± 1
DenseNet-201	Triplet	**20.8 ± 1**	9.9 ± 2	31.1 ± 1	11.0 ± 2	15.9 ± 2	22.3 ± 1
	P-NCA	20.2 ± 2	11.6 ± 3	28.4 ± 2	10.4 ± 1	16.0 ± 1	23.2 ± 3

**Table 5:** Model performance Feature Extractor loss optimal-dimension top layer. Bold indicates the best performing method, and grey highlights results that are not statistically significantly different from the best.

	MAP@R					
	Faces			Flanks		
Architecture	Lions	Chimps	Panda	Nyala	Zebra	Tiger
VGG-11	17.7 ± 1	**13.8 ± 3**	**32.0 ± 2**	**11.2 ± 0**	16.7 ± 2	22.8 ± 1
ResNet-18	19.0 ± 1	11.2 ± 2	26.3 ± 1	9.9 ± 2	**19.0 ± 0**	**24.6 ± 4**
DenseNet-201	**20.8 ± 1**	11.6 ± 3	31.1 ± 1	11.0 ± 2	16.0 ± 1	23.2 ± 3

## Data Availability

Zebra data publicly available at: https://code.google.com/archive/search?q=stripes%20dataset (accessed on 12 November 2019); Chimpanzees data publicly available at http://www.inf-cv.uni-jena.de/chimpanzee_faces.html (accessed on 12 November 2019); Tiger data publicly available at https://cvwc2019.github.io/challenge.html (accessed on 11 June 2021); Panda data available on request from https://researchdata.ntu.edu.sg/ (accessed on 16 May 2021); Lion data publicly available at https://github.com/tvanzyl/wildlife_reidentification (accessed on 29 October 2019); Nyala data publicly available at https://github.com/tvanzyl/wildlife_reidentification (accessed on 24 October 2019).

## Abbreviations

The following abbreviations are used in this manuscript:

DCNN	Deep Convolutional Neural Network
P-NCA	Proxy Neighbourhood Component Analysis
Proxy-NCA	Proxy Neighbourhood Component Analysis
MAP	Mean Average Precision
